# Modulation of Brain Kynurenic Acid by N-Acetylcysteine Prevents Cognitive Impairment and Muscular Weakness Induced by Cisplatin in Female Rats

**DOI:** 10.3390/cells13231989

**Published:** 2024-12-02

**Authors:** Teminijesu Dorcas Aremu, Daniela Ramírez Ortega, Tonali Blanco Ayala, Dinora Fabiola González Esquivel, Benjamín Pineda, Gonzalo Pérez de la Cruz, Alelí Salazar, Itamar Flores, Karla F. Meza-Sosa, Laura Sánchez Chapul, Edgar Rangel-López, Saúl Gómez-Manzo, Adrián Márquez Navarro, Gabriel Roldán Roldán, Verónica Pérez de la Cruz

**Affiliations:** 1 Doctorado en Ciencias Biológicas, Centro Tlaxcala Biología de la Conducta, Universidad Autónoma de Tlaxcala, Tlaxcala 90070, Mexico; ojuadeteminijesu@gmail.com; 2 Neurochemistry and Behavior Laboratory, National Institute of Neurology and Neurosurgery “Manuel Velasco Suárez”, Mexico City 14269, Mexico; danielaramirez@innn.edu.mx (D.R.O.); tblanco@innn.edu.mx (T.B.A.); dinora.gonzalez@innn.edu.mx (D.F.G.E.); karla.meza@innn.edu.mx (K.F.M.-S.); 3 Laboratorio de Neurobiología Conductual, Departamento de Fisiología, Facultad de Medicina, Universidad Nacional Autónoma de México, Mexico City 04510, Mexico; 4 Neuroimmunology Laboratory, National Institute of Neurology and Neurosurgery “Manuel Velasco Suárez”, Mexico City 14269, Mexico; benjamin.pineda@innn.edu.mx (B.P.); aleli.salazar@innn.edu.mx (A.S.); iflores1903@alumno.ipn.mx (I.F.); amarquez@innn.edu.mx (A.M.N.); 5 Department of Mathematics, Faculty of Sciences, Universidad Nacional Autónoma de México, Mexico City 04510, Mexico; gonzalo.perez@ciencias.unam.mx; 6 Departamento de Inmunología, Escuela Nacional de Ciencias Biológicas, Instituto Politécnico Nacional, Manuel Carpio, Plutarco Elías Calles, Miguel Hidalgo, Mexico City 11350, Mexico; 7 Neuromuscular Diseases Laboratory, National Institute of Rehabilitation “Luis Guillermo Ibarra Ibarra”, Mexico City 14389, Mexico; lausanchez@inr.gob.mx; 8 Cell Reprogramming Laboratory, National Institute of Neurology and Neurosurgery “Manuel Velasco Suárez”, Mexico City 14269, Mexico; edgar.rangel@innn.edu.mx; 9 Laboratorio de Bioquímica Genética, Instituto Nacional de Pediatría, Secretaría de Salud, México City 04530, Mexico; saulmanzo@ciencias.unam.mx

**Keywords:** chemo-brain, cognitive impairment, kynurenic acid, redox environment

## Abstract

Cisplatin (CIS) is a potent chemotherapeutic agent primarily used to treat hematologic malignancies and solid tumors, including lymphomas, sarcomas, and some carcinomas. Patients receiving this treatment for tumors outside the nervous system develop cognitive impairment. Alterations in the kynurenine pathway (KP) following CIS treatment suggest that certain KP metabolites may cross the blood–brain barrier, leading to increased production of the neuromodulator kynurenic acid (KYNA), which is associated with cognitive impairment. This study aimed to evaluate the effects of modulating brain KYNA levels by the administration of N-acetylcysteine (NAC), an inhibitor of kynurenine aminotransferase II (KATII), an enzyme responsible for KYNA biosynthesis on the cognitive and neuromuscular deficits induced by CIS. Female Wistar rats were divided into four groups: control, NAC (300 mg/day/8 days), CIS (3 mg/kg i.p/5 days), and NAC + CIS (both treatments co-administered in parallel). Seven days after the last CIS administration, cognitive performance, muscle strength, brain KYNA levels, KATII activity, and brain tissue redox profile (lipid peroxidation and oxidized/reduced glutathione (GSH/GSSG) ratio) were assessed. CIS did not affect short-term memory but induced long-term memory deficits and reduced muscle strength, effects which were prevented by NAC co-administration. CIS decreased the GSH/GSSG ratio and the number of cells in the brain cortex while it increased lipid peroxidation, KYNA levels, and marginal KATII activity. All these effects were attenuated by the co-administration of NAC. These findings suggest that NAC mitigates the side effects of CIS, such as chemo-brain and muscle weakness, by improving the redox imbalance and modulating KYNA levels by limiting its non-enzymatic production by reactive oxygen species (ROS).

## 1. Introduction

The World Health Organization estimated that in 2022, there will be 20 million new cancer cases worldwide, with around 10 million deaths from the disease. By 2050, the burden of cancer is projected to increase by approximately 61%, further impacting health systems, patients, and their families [1,2]. Cancer is characterized by the uncontrollable growth and spread of abnormal cells, which can result in death. The main current strategies for cancer treatment include chemotherapy, radiotherapy, and surgery [3].

Chemotherapy involves the use of drugs to destroy cancer cells and is a crucial component of cancer treatment aimed at improving survival rates. Unfortunately, these drugs are associated with multiple side effects, such as fatigue, nausea, sleep disorders, hormonal changes, mood swings, peripheral neuropathies, and organ toxicity [4]. Patients who have completed CIS chemotherapy often show behavioral changes and impaired memory functions. One of the most adverse side effects is chemotherapy-induced cognitive impairment, also known as the “chemo-brain effect”, which is characterized by attention deficits, lack of concentration, decreased processing speed, and working memory impairments. Chemo-brain can persist long after the treatment has ended, affecting up to 75% of patients [5,6,7]. Chemo-brain etiology is multifactorial, as it may result from inflammation, free radicals generation, and blood–brain barrier (BBB) disruption, leading to alterations in the normal processing of neuronal cells due to biochemical changes, as well as disproportionate production of metabolites that could promote neurotoxicity [8].

Cisplatin (CIS) is a platinum-based antineoplastic drug widely used in the treatment of solid tumors, including cervical, head, neck, prostate, breast, lung, testicular, and ovarian cancers [9,10]. As an alkylating agent, CIS causes DNA damage, resulting in cancer cell death. While effective, CIS is also associated with side effects, including nausea, weight loss, muscle atrophy, fatigue, and the chemo-brain effect [10,11,12]. Studies in rodents have shown that CIS induces thermal sensitivity, memory impairment, anxious behaviors, and muscle weakness [13,14]. Morphologically, CIS also affects white matter integrity in the prefrontal cortex and temporal lobe, two areas important for cognitive functions. Furthermore, CIS-induced cognitive impairment appears to involve the N-methyl-D-aspartate receptor (NMDAr) [15,16,17].

Recently, tryptophan catabolism has been linked to CIS-induced kidney injury, which increases peripheral and central kynurenine (KYN) levels [18]. In recent years, tryptophan catabolism through the kynurenine pathway (KP) has gained great attention in cancer research because it produces various metabolites with redox and immunosuppressive and neuromodulatory properties [19,20]. In the brain, kynurenic acid (KYNA), a major KP metabolite with neuromodulatory properties, is mainly produced in astrocytes by the kynurenine aminotransferase II (KATII) enzyme; it is also expressed, although to a lesser extent, in neurons [21]. Four isoforms (KATI-IV) of KATs, which use the precursor kynurenine (KYN) as a donor for an amino group, have been identified in humans and rodents. Among these, KAT II exhibits the highest enzymatic activity under physiological conditions and is therefore considered the primary biosynthetic enzyme responsible for brain KYNA production [22,23]. The major central pool of KYNA is synthesized locally in the brain from KYN, as KYNA itself cannot cross the blood–brain barrier (BBB). In contrast, KYN is predominantly produced in peripheral tissues and can cross the BBB [23]. This property of KYN facilitates alternative, non-enzymatic pathways for KYNA production. For example, KYN can be directly converted into KYNA through chemical interactions with reactive oxygen species (ROS) [24,25]. This non-enzymatic pathway becomes particularly significant in conditions involving an altered redox environment or compromised antioxidant systems. Thus, fluctuations in brain KYNA levels can be attributed to both enzymatic synthesis via KAT II and alternative ROS-mediated pathways, highlighting the dual mechanisms contributing to KYNA regulation under various physiological and pathological contexts.

KYNA, an antagonist of both the NMDAr and the α7-nicotinic receptors, influences extracellular levels of neurotransmitters such as glutamate, dopamine, GABA, and acetylcholine [26,27,28,29,30]. Elevated brain KYNA impairs learning, working memory, and cognitive flexibility, while the cerebral reduction in KYNA—whether pharmacological or through genetic models—improves cognitive function, suggesting that it is associated with pro-cognitive effects [31,32,33]. Considering the involvement of NMDAr in chemo-brain, KYNA may be a key mediator of CIS-induced cognitive deficit as it is implicated in neurological disorders, including depression, schizophrenia, amyotrophic lateral sclerosis, and Alzheimer’s disease [34,35].

N-acetylcysteine (NAC) is an antioxidant molecule used as an adjuvant in the treatment of neurodegenerative diseases and mental health problems (bipolar disorder, schizophrenia spectrum disorders, depressive disorders, anxiety disorders, etc.) [36,37,38]. NAC also inhibits KATII, the main enzyme responsible for KYNA production in the central nervous system (CNS) [39]. Based on this background, we aimed to evaluate the role of KYNA in cognitive alterations induced by CIS and whether NAC could mitigate these effects by modulating brain KYNA levels. This study offers insights into NAC’s potential as an adjuvant to prevent cognitive deficits during chemotherapy.

## 2. Materials and Methods

### 2.1. Materials

CIS was obtained from Accord Farma (Mexico) and was prepared in a saline solution vehicle (10 mg/10 mL). NAC, pyruvate, pyridoxal-5-phosphate (P5P), glutathione reduced form (GSH), oxidized glutathione (GSSG), O-phtaldehyde (OPA), N-Ethylmaleimide (NEM), diethylenetriamine pentaacetate (DTPA), glucose-6-phosphate (G6P), glucose-6-phosphate dehydrogenase (G6PDH) were obtained from Sigma-Aldrich Company (St. Louis, MO, USA). All other chemicals had the highest commercially available purity. Solutions were prepared using deionized water obtained from a Milli-Q (Millipore, Burlington, MA, USA) purifier system.

### 2.2. Animals

Female Wistar rats (200–260 g) were used. Animals were housed in groups of five in acrylic cages with ad libitum access to standard rodent diet and water. They were maintained under controlled conditions of temperature (25 ± 3 °C), humidity (50 ± 10%), and a 12 h light/dark cycle. Tissues were collected by decapitation, immediately dissected on ice, and preserved for a limited time at −70 °C. All experimental procedures followed the guidelines of the Guide for the Care and Use of Laboratory Animals (CICUAL-INNN), as well as the ethical standards of the Ministry of Health of Mexico (Authorized Protocol 31-24).

### 2.3. CIS and NAC Administration

Female rats were randomly assigned to 4 groups: (1) control, which received 0.9% NaCl (saline solution) intraperitoneally (i.p.) for 5 days along with a standard rodent diet; (2) CIS, which received 3 mg/kg cisplatin (i.p.) for 5 days; (3) NAC, which received 300 mg/day of NAC in pellets for 8 days; (4) NAC + CIS, where NAC was administered 3 days before and concurrently with 5 days of CIS treatment, totaling 8 days of NAC administration. Each group used for behavioral and strength testing consisted of 10 rats. Additionally, 14 animals were included in both the control and the CIS groups to obtain brain tissue 24 h after the last CIS administration. This additional tissue was used to evaluate the redox environment, peripheral KYN, brain KATII activity, KYNA levels, and the redox environment.

### 2.4. Novel Object Recognition Test (NORT)

Behavioral assessments were conducted between days 11 and 15 after the first CIS administration, in a schedule between 8:00 and 15:00 h. Rats were randomly assigned to groups and evaluated blindly. The NORT assessed learning and memory based on the natural tendency of rodents to explore novel objects. Rats were habituated to a 100 × 100 cm box for 10 min per day over two consecutive days. The test consisted of three phases: (1) In the training phase, two identical objects (A and A′) were placed diagonally and equidistant from the center of the box, and rats were allowed to explore them for 5 min. (2) Short-term memory (STM) was tested one hour later by replacing one object with a novel object B, and rats were reintroduced into the box and allowed to explore for another 5 min. (3) Long-term memory (LTM) was assessed 24 h later, replacing object B with another novel object C and rats were reintroduced into the box and allowed to explore for 5 min. Exploration time for each object was recorded, and the discrimination index (%) was calculated as [Time exploring novel object/Total exploration time] × 100. Video recordings were analyzed using the Solomon Coder beta 19.08.02 tracking software.

### 2.5. Wire Grip Strength Test

This test assessed grip strength and muscle weakness using a modified version of Ruan and Yao (2020) [40]. An iron bar (3 mm diameter, 50 cm length) was placed horizontally at 50 cm of height. Rats were placed on the bar to support their own weight with their forelimbs, and wood shavings were used underneath to prevent injuries from falling. The time spent hanging on the wire (latency) was recorded using a stopwatch, with three trials separated by 1 min intervals. The average latency was calculated as the final measure.

### 2.6. Biochemical Analysis

Biochemical assays were performed on cerebral cortex tissue and serum in two blocks: (1) 24 h after the last CIS administration (control and CIS groups) and (2) 24 h after behavioral testing (all groups).

#### 2.6.1. Lipid Peroxidation (LP)

LP was measured using thiobarbituric acid-reactive substances (TBA-RS). Brain tissue was sonicated in 500 µL of Krebs buffer (119 mM NaCl, 5 mM KCl, 2 mM CaCl_2_, 1.2 mM MgSO_4_, 5 mM glucose, 13 mM NaH_2_PO _4_ and 3 mM Na_2_HPO_4_, pH 7.4). Cortex homogenates (150 µL) were mixed with 250 µL of TBA solution (0.375 g of thiobarbituric acid + 15 g of trichloroacetic acid (TCA) + 2.54 mL of HCl in 100 mL) and incubated in boiling water for 15 min, then cooled on ice and centrifuged at 9800× *g* for 15 min. The optical density of the supernatant was read at 532 nm using a Synergy H1 microplate reader (Biotek-Agilent, Santa Clara, CA, USA). The results were expressed as micromoles of malondialdehyde per milligram of protein.

#### 2.6.2. GSH/GSSG Ratio

Brain cortex was homogenized (1:10, p/v) in Buffer A (154 mM KCl, 5 mM DTPA, and 0.1 M potassium phosphate buffer; pH 6.8) and mixed 1:1 with Buffer B (20 mM ascorbic acid, 10 mM DTPA, 40 mM HCl, and 10% trichloroacetic acid). Samples were centrifugated at 14,000× *g* for 10 min, and supernatants were filtered (0.22 µm). For GSH determination, samples were treated with OPA according to 38136155. For GSSG detection, GSH was neutralized with NEM (7.5 mM), reduced to GSH using sodium hydrosulfite (100 mM), and measured as an isoindole when OPA was added. Products were measured by fluorescence at 370 nm excitation/420 nm emission in a Synergy H1 microplate reader (Biotek-Agilent, Santa Clara, CA, USA). The GSH/GSSG ratio was calculated for each sample.

#### 2.6.3. Kynurenine (KYN) Determination

KYN determination was made using high-performance liquid chromatography (HPLC). Briefly, 100 µL of serum was mixed with 50 µL of perchloric acid (PCA, 6%) and centrifuged for 10 min at 14,000× *g*. An amount of 20 µL of each sample was eluted in C18 reverse-phase column (Shimadzu Nexcol C18 5 µm, 50 mm × 3.0 mm, Shimadzu, Tokyo, Japan) and L-KYN levels were determined in a RF-20Axs Shimadzu fluorescence detector (Shimadzu, Tokyo, Japan) at 365 nm excitation and 480 nm emission with a 50 mM sodium acetate, 250 mM zinc acetate, 2% acetonitrile, pH 6.2 mobile phase. The retention time of L-KYN was ~5 min, and results were expressed as pmoles of KYN/µL.

#### 2.6.4. Kynurenic Acid (KYNA) Determination

To quantify KYNA levels, tissues were homogenized in deionized water (1:10, *w*/*v*) and deproteinized with 30 µL of PCA; serum samples (100 µL were deproteinized with 50 µL of PCA), and finally, centrifuged at 14,000× *g* for 10 min. KYNA levels were analyzed using liquid chromatography with fluorescence detection (344 nm excitation and 398 nm emission) in an RF-20Axs Shimadzu fluorescence detector (Shimadzu, Tokyo, Japan). Samples (10 µL) were injected into a C18 reverse-phase column (Shimadzu Nexcol C18 5 µm, 50 mm × 3.0 mm; Shimadzu, Tokyo, Japan) and eluted with a mobile phase (50 mM sodium acetate, 250 mM zinc acetate, 2% acetonitrile, pH 6.2). KYNA had a retention time of ~7 min, and the results were expressed as fmoles of KYNA/mg of protein or fmoles of KYNA/µL.

#### 2.6.5. Kynurenine Aminotransferase II (KATII) Activity

KATII activity was measured in 100 µL of brain cortex homogenates (1:10 in homogenization buffer (Tris-base acetate buffer (0.5M, pH 8), pyridoxal phosphate (P5P, 50 mM), 2-mercaptoethanol (775 µL)) mixed with 100 µL of reaction cocktail (KYN (100 µM), pyruvate (1 mM), and P5P (80 µM) in Tris-acetate buffer (150 mM, pH 7.4)) and then incubated at 37 °C for 2 h in a shaking bath. After incubation, 20 µL of TCA (50%) and 1 mL of 0.1 M HCl were added to stop the reaction. Samples were centrifuged at 14,000× *g*, and KYNA was detected by fluorescence, as previously mentioned. The results are presented as pmoles of KATII activity/h/mg of protein.

#### 2.6.6. Protein Determination

Protein concentration was determined by the Lowry method using bovine serum albumin as a protein standard. Briefly, samples (10 µL) were mixed with C solution (1 mL of C solution, C = A solution (2% of Na_2_CO_3_, 0.4% of NaOH, and 0.2% of sodium tartrate), plus B solution (0.5% of Cu(SO_4_)_3_)) and incubated at room temperature for 10 min. Then, 100 µL of Folin solution (1:2 with deionized water) was added. Samples were incubated at room temperature for 30 min. The absorbance was determined at 550 nm in a Synergy H1 microplate reader (Biotek-Agilent, Santa Clara, CA, USA).

### 2.7. Immunohistochemistry

Twenty-four hours after the last behavioral test, rats were euthanized. Afterward, brains were dissected and coronally sectioned into two hemispheres. Each hemisphere was immersed in 4% paraformaldehyde at 4 °C for 1 h, cryoprotected in 30% sucrose solution for 24 h, frozen in liquid nitrogen–chilled isopentane, and stored at 70 °C until analysis. Eight-micron thick brain cryosections were mounted on slides and then blocked with 5% bovine serum albumin in phosphate buffer solution (PBS) at room temperature for 1 h. Then, samples were incubated with the next primary antibodies: mouse anti-NeuN (diluted 1:300; Abcam, Cambridge, UK) and rabbit anti-GFAP (diluted 1:250; Sigma Aldrich, St. Saint, USA) at 4 °C overnight. After washing three times with PBS, the sections were incubated with corresponding secondary antibody Cy3 conjugated goat anti-mouse IgG (diluted 1:250; Jackson ImmunoResearch Laboratories, Pike Country, USA) and Alexa Fluor 488 conjugated anti-rabbit IgG (diluted 1:300; Jackson ImmunoResearch Laboratories, USA) at room temperature for 1 h. After washing three times with PBS, sections were mounted on Vectashield antifade mounting medium with DAPI (Vector Laboratories, Burlingame, USA) and observed under a fluorescence microscope (Axio Examiner.A1, Zeiss, Brighton, US) using a 20x objective.

### 2.8. Statistical Analysis

Data were expressed as mean ± standard error of the mean (SEM). Comparations between two groups of independent samples were performed using the Mann–Whitney U test, and for more groups, the Kruskal–Wallis test with Dunn’s tests for multiple pairwise comparisons. The Friedman test was used to assess differences between three or more paired groups, and Wilcoxon signed-rank tests were used for multiple pairwise comparisons. Spearman correlation was used to analyze the association between variables. Significance was set at *p* < 0.05, using GraphPad Prism 7 (GraphPad, San Diego, CA, USA).

## 3. Results

Previous studies have reported that CIS administration alters the redox environment, reduces body weight, induces cognitive impairment, and stimulates tryptophan degradation in the periphery of rodents [18,41,42,43,44]. Therefore, we aimed to confirm whether the CIS dosing scheme used in this study was sufficient to induce changes in the redox environment, KYNA levels, and KAT II activity before proceeding with behavioral tests that could influence brain plasticity. After confirming CIS-induced alterations, we then assessed the effect of KYNA modulation by NAC administration on CIS-induced neurotoxicity.

### 3.1. Effect of Sub-Chronic CIS Administration on Redox Environment and Brain KYNA Levels in Female Rats

After the last administration of CIS, serum levels of KYN (KYNA precursor) and KYNA were determined (Table 1). The results showed approximately a 40% increase in serum KYN levels compared to the control, while serum KYNA levels decreased by 36%. Interestingly, brain KYNA levels increased more than twofold following CIS administration, despite the activity of KATII, the main enzyme responsible for KYNA production, which remained unchanged. When the brain redox environment was determined using the GSH/GSSG ratio, a significant decrease in this ratio was observed after CIS administration, indicating an oxidative shift compared to the control. These findings align with previous studies reporting elevated serum KYN levels after CIS treatment. More importantly, they highlight notable alterations in brain KYNA levels. 

### 3.2. Changes in Body Weight Induced by CIS and NAC Administration

**Figure 1:**
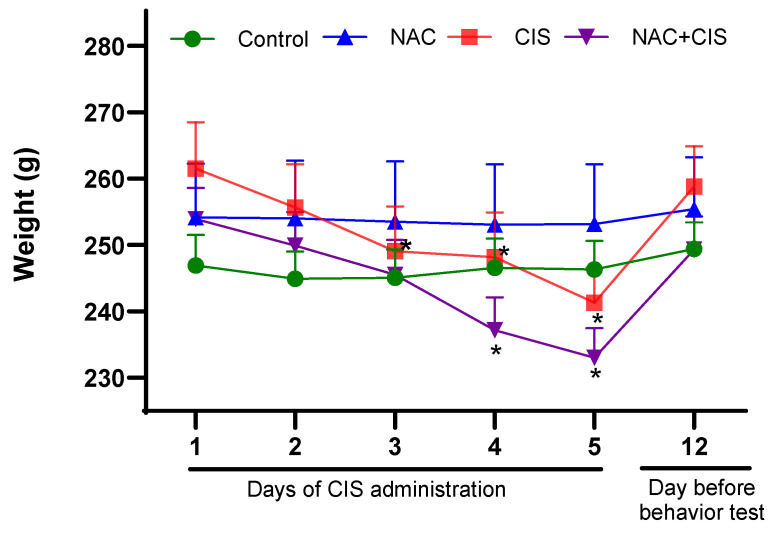
Changes in body weight of rats administered with CIS and NAC. The body weight (g) of rats was monitored from the beginning of CIS administration until one day before the start of cognitive testing. The experimental groups included control (0.9% saline), CIS (3 mg/kg/5 days), NAC (300 mg/day/8 days), and NAC + CIS (NAC for 8 days, with concurrent CIS administration from days 3 to 7). Data are represented as the mean ± SEM of 10 animals per group. * *p* < 0.01 vs. the initial body weight within each group, based on the Friedman test with Wilcoxon signed-rank test for multiple pairwise comparisons.

After confirming that sub-chronic administration of CIS induced alterations in brain KYNA levels, our next step was to modulate KYNA using NAC—an inhibitor of KATII- to determine whether KYNA is implicated in the cognitive impairment induced by this chemotherapeutic agent. Body weight was monitored during CIS administration and one day before behavioral tests across all groups to assess the potential effects of treatments on the general condition of the rats. As shown in Figure 1, the control and NAC groups did not exhibit significant changes in body weight over time. In contrast, rats from the CIS group showed a reduction in body weight from day 3 to 5 of treatment, with a decrease of ~4.5–8% of their initial weight. A similar pattern was observed in the NAC + CIS group, in which body weight decreased by 6.6% and 8.2% on days 4 and 5, respectively, compared to initial weight. Both the CIS and NAC + CIS groups regained body weight seven days after the final administration of CIS and one day before the behavioral and strength tests began.

### 3.3. Effect of Sub-Chronic Administration of CIS on NORT in Female Rats

**Figure 2:**
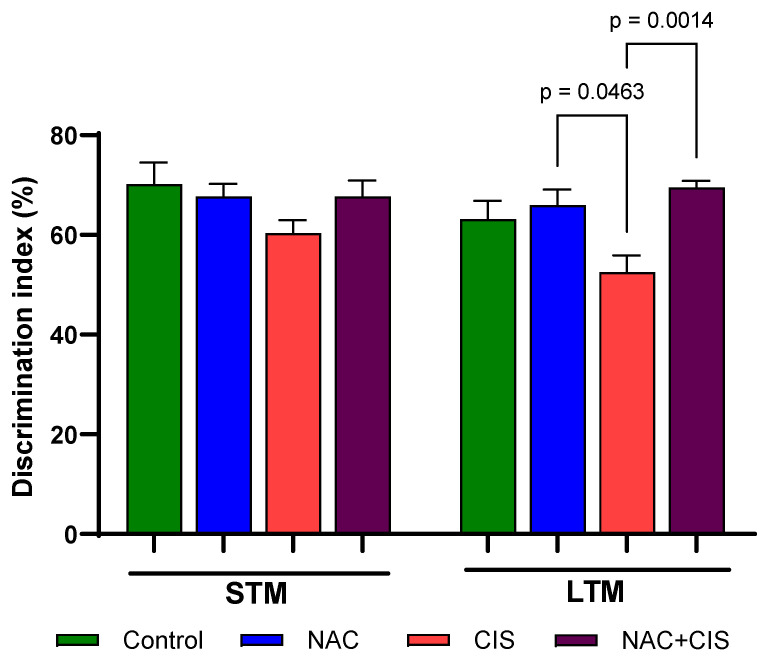
Effect of N-acetylcysteine (NAC) administration on short-term memory (STM) and long-term memory (LTM) impairments induced by sub-chronic cisplatin (CIS) administration. Cognitive performance was assessed using the novel object recognition test (B: a novel object in STM; C: a novel object in LTM) 7 days after the last CIS administration. The recognition index was calculated to evaluate STM and LTM using 9–10 animals per group. Data are shown as the mean ± SEM, based on the Kruskal–Wallis test followed by Dunn’s test.

To evaluate the impact of KYNA modulation on the cognitive impairment induced by sub-chronic CIS administration, we assessed short-term memory (STM) and long-term memory (LTM) using the NORT. The discrimination index calculated as the time spent exploring the novel object relative to the total exploration time was used to evaluate memory performance (Figure 2). The results showed no significant alterations in STM across the experimental groups, although the CIS group showed a trend toward a lower discrimination index compared to the control group. In contrast, when LTM was evaluated, CIS significantly reduced the discrimination index compared to the control group (~17% vs. control). However, this reduction was significantly attenuated when NAC was co-administrated with CIS, suggesting a protective effect of NAC against the cognitive impairment induced by CIS.

### 3.4. Effect of NAC and CIS Co-Administration on Muscle Grip Strength in Female Rats

**Figure 3:**
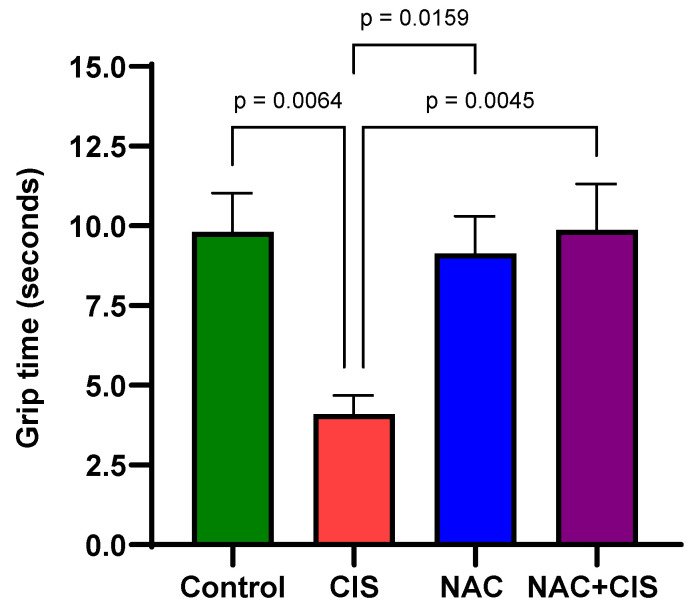
Effect of N-acetylcysteine (NAC) administration on muscle performance induced by sub-chronic administration of cisplatin (CIS). Grip strength was calculated as the average time of three different trials separated by 1 min for each animal per group (n = 10). Data are represented as the mean ± SEM, based on the Kruskal–Wallis test followed by Dunn’s test.

A known side effect of CIS treatment is skeletal muscle dysfunction. To evaluate this, we assessed grip strength as an indicator of muscle performance (Figure 3). The results showed that CIS reduced the grip strength by approximately 50% compared to the control group (4.1 ± 0.6 vs. 9.8 ± 1.2 s, respectively). However, co-administration of NAC with CIS completely prevented this reduction. In fact, the NAC + CIS group displayed grip strength comparable to that of the control group (9.9 ± 1.5 s). Notably, this effect was independent of body weight, as all animals across groups had similar weights at the time of the test, as shown in Figure 1.

### 3.5. Effect of NAC on CIS-Induced Brain KYNA Levels Alterations

**Figure 4:**
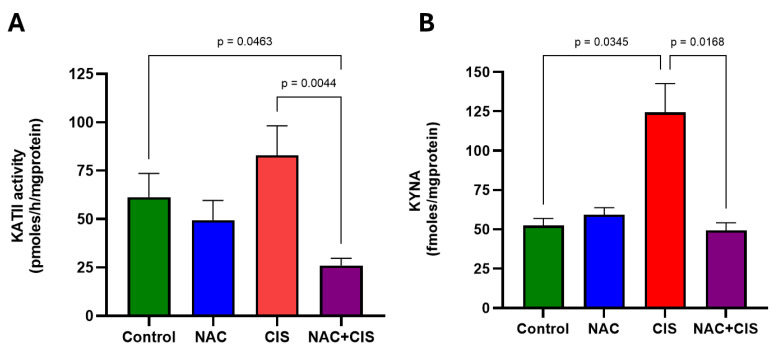
Effect of simultaneous administration of NAC and CIS on KATII activity and KYNA levels. The brain cortex was analyzed to evaluate KATII activity (**A**) and KYNA levels (**B**). Data represented as the mean ± SEM of 7 animals per group. Based on the Kruskal–Wallis test, with Dunn’s test for multiple pairwise comparisons.

Having demonstrated that the deleterious effects of CIS administration on long-term memory and muscle strength can be prevented by NAC, we next investigated whether the protective effect of NAC is mediated through the modulation of KYNA levels. First, we assessed KAT II activity, the primary enzyme responsible for KYNA production in the brain (see Figure 4A). The results showed no significant changes in KATII activity in the NAC and CIS groups compared to the control group. However, the combination of NAC + CIS significantly reduced KATII activity compared to control and CIS groups (26 ± 4 pmoles/h/mg protein vs. 61 ± 12 or 83 ± 15.2 pmoles/h/mg protein, respectively). Although no changes in KATII activity were observed in the CIS group, CIS administration significantly increased brain KYNA levels compared to control (124.3 ± 18.3 vs. 52.4 ± 4.5 fmoles/mg protein, respectively) (see Figure 4B). This KYNA increase was prevented when NAC was co-administrated with CIS, suggesting that the protective effect of NAC might be mediated by the modulation of brain KYNA levels.

### 3.6. Effect of NAC Co-Administration on the Oxidative Environment Induced by CIS

**Figure 5:**
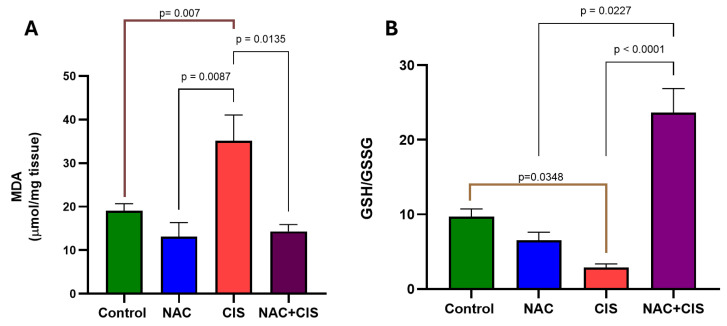
Effect of co-administration of CIS and N-acetylcysteine (NAC) on lipid peroxidation (**A**) and the GSH/GSSG ratio (**B**) induced by sub-chronic administration of cisplatin. The brain cortex was used to evaluate the redox environment. Data are represented by the mean ± SEM of 7 animals per group. Based on the Kruskal–Wallis test, followed by Dunn’s tests for multiple pairwise comparisons (in brown when comparing only vs. control).

Since KYNA can be produced not only by KATs but also through alternative non-enzymatic pathways involving reactive oxygen species (ROS) directly reacting with its precursor, KYN, and considering that CIS administration did not significantly alter KATII activity, we investigated whether CIS creates an oxidative environment that could promote this alternative KYNA production pathway. This cellular redox environment was evaluated by measuring malondialdehyde (MDA) levels (a final product of lipoperoxidation) and the GSH/GSSG ratio in the cerebral cortex (Figure 5A and Figure 5B, respectively). The results showed that CIS increased MDA levels while reducing the GSH/GSSG ratio compared to the control group (MDA: 35.1 ± 6 vs. 19.1 ± 1.6 µmoles/mg tissue; GSH/GSSG: 2.9 ± 0.5 vs. 9.7 ± 1.0), indicating an oxidative environment is ideal for the alternative production of KYNA. However, when NAC was co-administrated with CIS, it significantly reduced lipid peroxidation, as evidenced by lower MDA levels, and restored the GSH/GSSG ratio. Remarkably, the GSH/GSSG ratio not only returned to control levels but doubled compared to the control group, suggesting a robust antioxidant effect of NAC.

### 3.7. Correlation Between Long-Term Memory or Muscle Grip Strength and KYNA Levels

**Figure 6:**
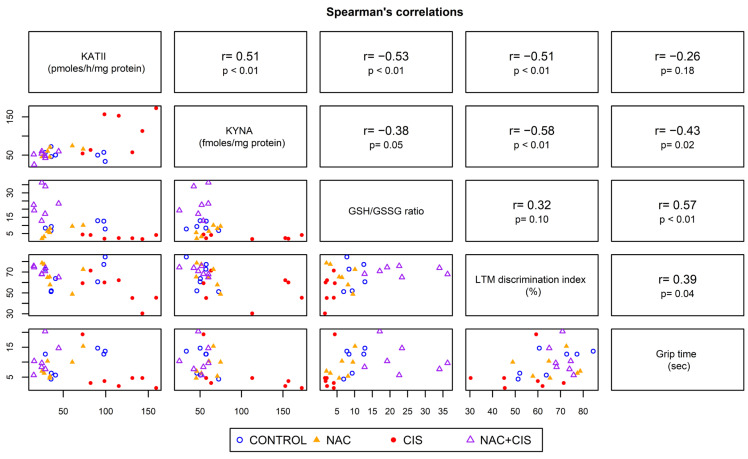
Correlation between KYNA levels, KATII activity, the GSH/GSSG ratio, long-term memory, and muscle strength. The matrix in the lower left quadrant contains the scatter plot of all parameters, and the upper right quadrant contains Spearman’s coefficient (r) and is associated with a *p*-value (n = 7, per group).

After confirming the protective effect of KYNA modulation through NAC administration, we investigated to determine whether there was a direct relationship between cognitive alterations, muscle weakness, brain KYNA levels, and redox environment. Spearman’s correlations were calculated to explore these relationships (Figure 6). The results revealed a negative correlation between long-term memory performance (as measured by the discrimination index in NORT) and both brain KYNA levels and KATII activity. This indicates that a higher discrimination index (reflecting better cognitive performance) is associated with lower KYNA levels and reduced KATII activity. These findings align with the observed negative correlation between brain KYNA levels and grip strength, as well as the positive correlation between grip strength and the GSH/GSSG ratio. This suggests that lower KYNA levels are associated with improved muscle strength. Interestingly, KYNA levels and KATII negatively correlated with the GSH/GSSG ratio, indicating that increased KYNA production is linked to an oxidative environment. Furthermore, the GSH/GSSG ratio positively correlated with the discrimination index in NORT. These data suggest that the effect of KYNA on cognitive performance is closely related to a redox environment.

### 3.8. Effect of N-Acetylcysteine Co-Administration on Cerebral Cortex Cellular Density and Composition Induced by Sub-Chronic Administration of CIS

**Figure 7:**
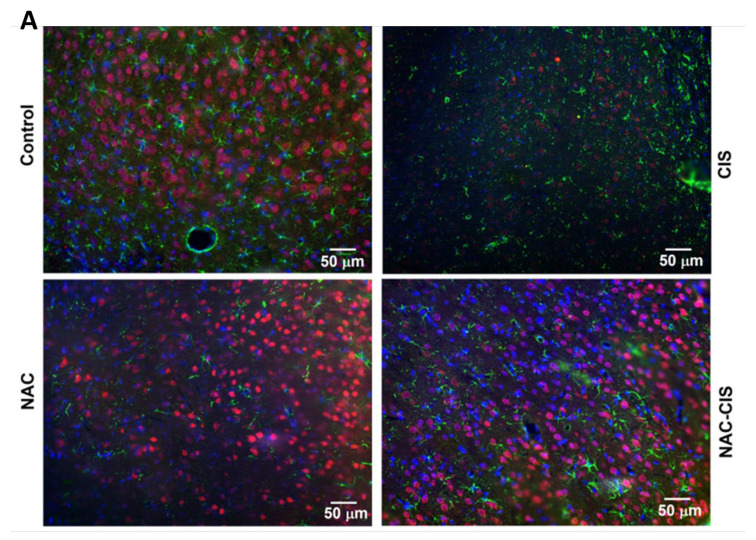
Effect of N-acetylcysteine and cisplatin co-administration on the number of astrocytes and neurons in the cerebral cortex. (**A**) Representative images show immunofluorescence detection of neurons (NeuN, red), astrocytes (GFAP, green), and nuclei (DAPI, blue) within the cerebral cortex across the four experimental groups. Images were captured at 20× magnification.

Finally, we wondered whether NAC administration could preserve brain cell populations in the cerebral cortex (Figure 7). We found that CIS induced a reduction in the number of astrocytes and neurons in cortical tissue compared to the control group. However, the co-administration of NAC effectively prevented this reduction, preserving the cellular density and composition of the cerebral cortex despite CIS treatment.

## 4. Discussion

CIS is known to be toxic to the nervous system [45]. As mentioned earlier, CIS neurotoxicity also promotes behavioral changes and cognitive deficits. International regulations for establishing the dose of cisplatin during chemotherapy are limited by its ability to induce neurotoxicity [46]. Although several alternatives have been proposed to maximize the efficacy of CIS while reducing its neurotoxic effects, such as co-administration of chemoprotective or rescue therapies, satisfactory results have been limited.

CIS disturbs neuronal signaling and alters long-term potentiation (LTP), particularly by disrupting glutamatergic neurotransmission [47]. Endogenous metabolites produced from the tryptophan catabolism, such as KYNA, are well-known to modulate glutamatergic neurotransmission by antagonizing NMDAr. Thus, elevated KYNA levels can induce hypofunction in these receptors. This is the first study exploring the role of KYNA on the chemo-brain effect induced by the sub-chronic administration of CIS. Chemo-brain refers to the cognitive impairment that arises from chemotherapy, which may persist long after treatment is discontinued. A study by Simovic and coworkers provided key evidence suggesting the potential involvement of KYNA in this effect, as they reported a three-fold increase in peripheral KYN levels following CIS administration [18]. KYN is a metabolite of the tryptophan catabolism produced through the KP that can easily cross the BBB, thereby facilitating KYNA production within the CNS. It is important to note that KYNA cannot cross the BBB, making the peripheral increase in KYN directly contribute to raising brain KYNA levels. To confirm whether the previously documented peripheral increase in KYN promoted KYNA production under our conditions, we measured serum KYN and KYNA levels, brain KYNA levels and KATII activity, and the GSH/GSSG ratio 24 h after the last administration of CIS. As expected, peripheral KYN levels increased, while serum KYNA decreased after CIS-subchronic administration, indicating that the increased availability of KYN across the BBB induces KYNA production within the CNS. Interestingly, KATII activity remained unchanged after CIS administration, implying that brain KYNA elevation could be partially due to the ROS-activated alternative pathway of KYNA production. CIS also causes mitochondrial malfunction as a result of mitochondrial DNA (mtDNA) damage, causing reactive oxygen species (ROS), which exacerbates oxidative stress. The overproduction of ROS is a distinctive feature of chemo-brain, accompanied by a reduced antioxidant capacity. This idea is supported by the significant reduction in the GSH/GSSG ratio, an antioxidant marker, and the increase in MDA levels after CIS administration in the brain, suggesting that an oxidative environment promotes KYNA production via this alternative pathway.

As mentioned before, the increase in brain KYNA levels has been consistently linked to cognitive impairment, mainly due to KYNA’s antagonistic effects on NMDAr and α7-nicotinic receptors, both essential for learning and memory consolidation processes as well as for the modulation of glutamatergic, dopaminergic, and cholinergic neurotransmission. The next step was to determine whether the increase in brain KYNA was associated with cognitive dysfunction induced by CIS administration. Currently, there is no specific KATII inhibitor available for clinical use [48], and since our results also indicate that KYNA could be partially produced by an oxidative alternative pathway, we used NAC as a strategy to modulate brain KYNA levels. NAC not only inhibits KATII but also promotes an antioxidant environment, thereby addressing both the canonical and alternative pathways of KYNA production in the context of CIS treatment [24,33,39,49].

When cognitive function was assessed using the NORT, no significant changes were observed on STM one week after completion of CIS treatment. However, there was a trend among CIS-treated rats struggling to discriminate between the familiar and the novel object. These results align with previous studies reporting memory deficits after CIS administration [50,51]. In contrast, LTM impairment was completely prevented when NAC was co-administrated with CIS, suggesting that KYNA modulation by NAC may be involved in this effect. Moreover, CIS administration is also linked to decreased muscle strength in experimental models, and muscle atrophy is a known predictor of reduced survival in patients receiving chemotherapy [12,41,52,53]. In this study, CIS reduced grip strength time by more than 50%, suggesting the onset of muscle weakness. However, this reduction in grip strength time may also be related to the lack of motivation or depressive-like behavior previously reported for CIS administration [52,54,55,56]. Notably, when NAC was administrated concurrently with CIS, grip time remained equal to that of the control group, indicating that NAC effectively prevents CIS-induced muscle weakness and possibly demotivation [57]. Additionally, CIS induces neuropathy due to its propensity to accumulate in the dorsal root ganglia, so there it is possible that the neuroprotective capacity of NAC could prevent damage in this region [46].

Once cognitive impairments and muscle weakness induced by CIS were characterized, along with the protective effects of NAC, we further examined the underlying mechanism. We observed that the increase in brain KYNA following CIS administration was not completely associated with increased KATII activity. However, NAC supplementation restored basal brain KYNA levels and reduced KATII activity by more than 50%. As mentioned earlier, KYNA can be produced through a non-canonical ROS-induced pathway; thus, CIS-induced oxidative stress created an ideal environment for KYNA production through KYN interaction with ROS [24,58]. When NAC was co-administrated with CIS, brain KYNA levels decreased, and the redox environment shifted towards a more reductive state, as indicated by an increased GSH/GSSG ratio beyond baseline levels and the reduction in lipid peroxidation. These findings suggest that NAC effectively mitigates the oxidative stress induced by CIS, thereby preventing conditions that could facilitate alternative KYNA production. Additionally, NAC preserved brain cell integrity, contributing to the observed protection against CIS-induced alterations.

The pro-cognitive effects of NAC through KYNA modulation have also been observed in other experimental models, where NAC pre-administration reduces brain damage following adverse events and correlates with decreased brain KYNA levels [33,38]. Furthermore, the protective effects of NAC against CIS-induced toxicity have been documented in several studies, where NAC is able to improve testicular toxicity, mitigate premature senescence, attenuate cholinergic dysfunction, and reduce anxiety episodes in rodents [59,60]. However, this is the first study to specifically link the beneficial effects of NAC with KYNA modulation, as confirmed by Spearman’s correlation, showing a negative correlation between KYNA levels and both the LTM discrimination index in the NORT and grip strength. These results align with previous findings associating elevated brain KYNA levels with memory and depressive behaviors [32,61,62,63]. Notably, the cognitive impairment and muscle weakness observed after CIS administration are also observed in cancer patients who received this treatment [5,64]. Furthermore, the negative correlation between the GSH/GSSG ratio and brain KYNA levels suggests that the cellular redox environment directly influences KYNA production.

This study has some limitations. We did not assess whether NAC affects CIS metabolism or clearance nor whether KYNA is also involved in the chemo-brain effect induced by other chemotherapeutics. Moreover, it remains unclear at what stage of CIS treatment it would be more convenient to administer NAC, compromising the antineoplastic effect of CIS. Finally, it would be interesting to evaluate whether an immediate post-chemotherapy treatment with NAC could mitigate its side effects within the CNS. Despite these limitations, these findings may have immediate translational implications. Given the established safety profile of NAC, it could be considered as an adjuvant treatment alongside CIS to potentially reduce chemo-brain effects.

## 5. Conclusions

Our findings demonstrate that KYNA is implicated in sub-chronic CIS administration-induced cognitive impartment. Modulating brain KYNA levels with NAC effectively mitigates side effects of CIS, such as chemo-brain and muscle weakness. These results suggest that NAC could be a promising strategy to prevent CIS-induced neurotoxicity and muscle dysfunction in patients receiving this type of chemotherapy.

## Figures and Tables

**Table 1:** Changes in KP metabolites and redox environment after sub-chronic administration of CIS.

Experimental Group	Serum KYN (pmoles/µL)	Serum KYNA (fmoles/µL)	Brain KYNA (fmoles/mg Protein)	Brain KAT II Activity (fmoles/h/mg Protein)	Brain GSH/GSSG
Control	32.4 ± 2.5	897.0 ± 112	89.56 ± 6.65	230.2 ± 24.3	5.84 ± 0.67
Cisplatin	45.2 ± 4.5 *	567.3 ± 68.2 *	226.50 ± 37.21 *	241.2 ± 31.5	2.51 ± 0.48 *

Control: n = 6 and CIS: n = 8 rats. * *p* < 0.05 vs. control based on Mann–Whitney U test.

## Data Availability

Data used to support the findings of this study are available with the corresponding author upon request.
